# Rational Steps in the Scientific Treatment of Nervous Prostration, Headache and Neuralgia

**Published:** 1900-05

**Authors:** Ambrose L. Ranney

**Affiliations:** New York City


### BUFFALO MEDICAL JOURNAL.

VOL. XXXIX.—LV. MAY, 1900. No. 10.

### Original Communications.

### RATIONAL STEPS IN THE SCIENTIFIC TREATMENT OF NERVOUS PROSTRATION, HEADACHE AND NEURALGIA.

By AMBROSE L. RANNEY, M. D., New York City.

(*Concluded from April.*)

CASE V.—*Nervous prostration, with chronic insomnia and mental distress and confusion.*—W. C., aged 42, minister of the Gospel, married.

*Family History.*—Mother living, aged 84. Father was a delicate man and had headaches. One brother and one sister have severe headaches. One brother has several times broken down in his studies from eye-pain and asthenopia.

*Eye Defects.*—Myopic astigmatism, O.D.—1.25 c.; O.S.—0.50c. Exophoria 10°. Crossed diplopia of 4° (with red glass before either eye). No hyperphoria. Adduction, 18°. Abduction, 12°. Right sursumduction, 3°. Left sursumduction, 3°. Presbyopia (uses + 1.00 s. for reading).

*History of Case.*—This patient was never a very strong man and had always been a hard student. About six years ago he began to suffer from sleeplessness, confusion of thought, and an utter inability to apply himself to his work for more than a few minutes at a time. He put himself in the care of a prominent physician of this city, and at that time was examined by an oculist of note, who prescribed glasses to correct his astigmatism. These gave some temporary relief, but he soon broke down completely, resigned his position, and for many months was unable to do any work. After a long rest, he again took charge of a church, but his insomnia soon became so severe that an extended vacation was necessary. Since that time, by the most regular habits, careful diet, and daily exercise in the open air, he had been able to keep his position, although he feared a collapse at any moment. Finally, his loss of sleep, distress in his head (although he had no actual headache), and confusion of thought became so constant that he was again on the verge of resigning his position when he came to me for treatment.

*Treatment and Results.*—The treatment of this case consisted simply of two graduated tenotomies for the relief of the exophoria, and subsequently a reading-glass to correct his presbyopia. The result of this treatment was most gratifying. A report received from the patient within three weeks after the first tenotomy states that he "now sleeps well, and has great relief from the distress which he has so long experienced in his head."

For several years past he has been doing his work with comfort, and with no return of his old symptoms. He sleeps well, with the exception of an occasional poor night after an unusually trying day's work. He has taken no medicinal treatment.

During a visit at my office, within a few months, he stated that "he considered himself entirely cured of his old troubles."

Case VI.—*Sudden physical and nervous collapse, preceded by sleeplessness and mental distress.*—A. C. H., aged 46, manufacturer, married.

*Family History.*—Both parents lived to seventy-six years. Two paternal uncles died of phthisis. No hereditary tendency to nervous diseases.

*Eye Defects.*—Vision $\frac{20}{20}$ without atropine. Under atropine, a latent hypermetropia of +1.00 s. in each eye. Patient had never used a glass for reading. Esophoria, 5° (after using prismatic glasses for a short time, the patient showed esophoria of 13°). Adduction, 24°. Abduction, 4°+. Later on the adduction exceeded 50°, and the abduction fell to 0. Homonymous diplopia with the red glass over one eye was usually present, and at times without the red glass.

*History of Case.*—This patient had been a perfectly well man and had carried on a very large business up to fifteen years ago. At this time, while attending a sale in New York, he was suddenly seized with a dizziness, faintness, and a sore feeling in his head. These symptoms lasted for three years in spite of all treatment, during which time he suffered severely from sleeplessness, extreme nervousness, and soreness in his head. He was unable to look out of a car window while traveling without great distress. He had suffered all his life from obstinate constipation, and had taken cathartics so regularly that now any cathartic water causes intestinal hemorrhage. When this patient first came to me he was able, by the most careful diet, regular habits, and by retiring at eight or nine o'clock, to carry on his enormous business only with the greatest difficulty because of the following symptoms: inability to sleep at night, which at times was very distressing and persistent; extreme nervousness after the slightest fatigue; mental depression without any cause; hot flashes up and down his spine; pain in his shoulders and across his back. His insomnia was often prolonged and very exhausting after any slight excitement or fatigue.

*Treatment and Results.*—The treatment of this patient consisted at first of the wearing of prisms to relieve the esophoria, and later on of graduated tenotomies on both internal recti. Subsequently,

+0.50 s. glasses were given for constant wear, and +1.00 s. glasses for near work. The distance glasses he soon discarded, as they annoyed him greatly. The improvement in his condition was marked and continuous from the first. An extract from a letter received from him two months after the operation on his eyes speaks for itself. He says: "Seemingly I am all right, feeling better every day; have not had a headache for a month; appetite good and I sleep well." Over eight years have now elapsed without any return of his former ill health, during which time he has constantly been engaged in active business pursuits. During these eight years I have seen this patient frequently at my office. His eye-tests remain unchanged from those obtained after the last tenotomy, and are as nearly normal as it is possible to make them.

Case VII.—*Nervous prostration, headache, insomnia and confinement to bed for nearly one year.* —Mrs. W., aged 55. Married.

*Eye Defects.*—Hypermetropia, +1.75 s. Presbyopia (uses +4.50 s. for reading). Esophoria, 7°. Adduction, 23°. Abduction, 3°+. Later on she disclosed: Right hyperphoria, 3°; right sursumduction, 6°+; left sursumduction, 2°—.

*History of Case.*—This patient is the wife of a prominent physician, and, as such, has had the benefit of the best medical talent of the state in which she resides. She had always been a delicate woman up to the time when my professional opinion of the case was asked. For a year or more before I first saw her she had been a victim to nervous prostration and confined most of the time to her bed or room. Her life had been despaired of during this interval at times and the case seemed to present problems in diagnosis which puzzled the best medical men whom she had consulted. When she had gained sufficient strength to allow of her being moved with safety, her husband was advised to take her to a Southern climate. On her way to Florida he was advised to consult me in reference to the case, when he passed through New York.

When I first saw this patient she was in a state of extreme physical and mental depression, was unable to walk for even short distances without great fatigue, was sleepless and despondent, and was brought to my office in a carriage from a hotel not far from my residence.

*Treatment and Results.*—At the first visit prisms were given to relieve the esophoria, and in five days a graduated tenotomy was done on one internal rectus. The patient began to feel the benefit of this step from the first. The second day after the tenotomy she reported that she had walked a mile and a half—a thing she had not done for over a year. Five days after the first tenotomy, a second one was performed on the other internal rectus, prisms having been worn in the meantime. Two days following this the patient walked five miles, visited an art museum in the morning, and attended a theatre in the evening. In spite of the unusual fatigue and excitement, she was still sleeping well and feeling stronger than for many years. With the improvement of her general health came an entire cessation of an obstinate bladder trouble, which had given her annoyance for many years, and was probably due to her weak muscular and nervous condition. The pain in the bladder, which was probably of the neuralgic type, ceased after the relief of the eye tension, and has never returned.

After an interval of four months, during which she had been comparatively well, she returned to New York to complete her treatment. A high degree of hyperphoria was found, and prisms were combined with her hypermetropic glasses to relieve it. With these glasses the patient passed eight months of almost absolute freedom from distress of any kind, when a graduated tenotomy was performed and the hyperphoria prisms removed. She reported later that she was sleeping well, was able to attend to her household duties, could walk long distances, had taken no medicine for over a year, and was regarded by her husband and friends as restored to perfect health. No relapse of her old symptoms has ever occurred.

Case VIII.—*Constant headache for several years, and inability to use the eyes without greatly intensifying the pain.*—Miss F., single, aged 19. This young lady was brought to my office by her uncle, a physician, who had tried all remedies to relieve her headaches without any benefit. The pain was chiefly in the occipital region, and was constant both day and night. Whenever she tried to use her eyes, the pain became more intense. As she was desirous of becoming a teacher, her chance of being self-supporting in that field seemed far distant. She had been treated for the anemia that was very apparent; her organs had been examined and found to be free from disease; her bowels' action was normal; her menstruation regular, and no apparent cause existed to explain her long-continued suffering.

An examination of the eyes disclosed apparent emmetropia and she tolerated no plus-glasses, but under atropine a very low grade of hypermetropia was found. She was prescribed prisms for esophoria. At once she felt somewhat relieved, and she reported the next day that the glasses were very comforting to her. Within a month, I performed graduated tenotomies upon both the interni, as a high degree of "latent" esophoria manifested itself. Within a week after the last operation the pain in the head ceased, and the patient rapidly regained her strength, weight and color.

Several years have elapsed since then, yet this patient continues to be free from pain and has become a strong, robust, healthy woman. She has not yet worn glasses. Her ability to use her eyes without them and the entire disappearance of pain, leave no ground for me to prescribe them either for reading or constant use.

Case IX.—*Complete nervous prostration, loss of memory and inability to concentrate thought. Suspected softening of the brain.*—Mr. C., married, aged 42; accountant. This is one of the most remarkable and obscure eye-cases that I have ever yet encountered. The final results obtained were far better than any one interested in the case at first hoped for or expected. The eye factors in the case were peculiarly obscure, and harder to determine satisfactorily to myself than to correct surgically when once clearly shown. This case was in reality an "eye-puzzle"—even to one with a large experience in the solution of eye-problems.

The patient had suffered from severe headaches since boyhood; he had once had syphilis, to which most of his later troubles had been attributed by different physicians; he had a hereditary predisposition to insanity and consumption; he had for many years used his eyes and brain constantly for long hours each day; he had borne great anxieties and responsibilities in his business relations; finally he had collapsed mentally and physically and had been pronounced by a skilful diagnostician, prior to my seeing the patient, as suffering from softening of the brain.

His physical and mental breakdown was preceded for some months by insomnia, constant headache, extreme nervousness and debility. It was attributed by most physicians whom he consulted to his long hours, his great cares, and possibly to some remaining taint of specific trouble. No therapeutic measures proved of any avail and he was instructed to give up all business and to travel. He did so for some months. He returned home rather worse than improved. His constant headache, mental confusion, extreme physical weakness, and insomnia were not relieved.

This was the condition of the patient when I first examined him in my office. He had practically abandoned all drugs for some months prior to consulting me. To publish all the eye-records of this case would take more space than I am justified in giving to it here. In brief, it may be said that by lowering the right eye, raising the left, and relieving a high degree of abnormal tension upon both interni, the eyes were at last brought to a state of perfect adjustment. Perfect health was thus reëstablished without the use of a drug. The refraction of this patient consisted of a moderate degree of near-sightedness and astigmatism, for which he had worn and still wears glasses.

For the past eight years this patient has remained in good health, in spite of business cares and duties that would tax the strongest individual. He sleeps well, has the clearest of intellect, has little or no headache and considers himself as permanently cured by the correction of eye-strain.

Case X.—*Terrific neuralgic paroxysms that anesthetics alone could control.*—Miss W., single; aged 18. This patient was known throughout the region in which she resided as the "screaming girl." Her piercing shrieks when attacked with general neuralgia, that occurred very frequently, could be heard for long distances in the quiet of the village where she resided; and all the physicians who were consulted could find no remedies but morphine and anesthetics to control the paroxysms. She suffered constantly from headache. When I first saw her, her vision was so poor that she used her eyes but little, and wore glasses which had been previously ordered by an oculist. She had then some evidences of conical cornea, but could get $\frac{20}{70}$ vision with the glasses she was using. No modification of these glasses seemed to improve her vision in either eye. On testing her eye muscles, it was found that she *had both vertical* and *homonymous diplopia*, viz.: when a red glass was placed before either eye, and the patient was instructed to look at a candle twenty feet away, unconquerable double images appeared at different heights and the image of the right eye deviated to the right.

The surgical steps required in this case to correct the double type of cross-eye consisted in lowering the left eye, raising the right eye and afterward relieving the abnormal tension of both internal recti muscles. To do this several months were devoted by me to the case, with frequent intervals of absence and travel on the part of the patient between the various steps. Since this work was performed the neuralgia paroxysms have been entirely arrested, and have not returned for over seven years.

I could multiply these ten illustrative cases many times from my case-records, if I deemed it important to do so here. But as I have recorded in my recent work, "Eye-Strain in Health and Disease," many equally striking ones with a complete record of the eye-tests of each and the steps taken to correct them, I do not deem it necessary here to greatly enlarge the number.

I have shown in this paper how six hopeless cases of nervous prostration (four of whom had alarming mental symptoms as a complication) were restored to perfect health by the surgical relief of eye-strain after drugs had accomplished nothing. I have brought forward two cases of horribly acute neuralgic paroxysms that had puzzled many physicians of repute—yet who got well at once without drugs when their eyes were properly investigated. Finally, I have given to the reader two typical cases of incessant and apparently incurable headache—that were at once cured by eye-treatment alone.

The science of eye-treatment to-day is not what it was even a decade ago. The presence or absence of equilibrium (or perfect adjustment) of the ocular muscles can be scientifically measured today, as well as the exact arcs of rotation of each eye independently of its fellow, by instruments of precision that were not perfected or used ten years ago. It is absolutely essential today to accuracy of diagnosis that such instruments of precision be used, before any opinion is final. It is no longer creditable to anyone to hastily decide concerning the presence or absence of heterophoria[2] on one or two examinations. It is impossible in some cases to unearth and positively demonstrate "latent" heterophoria without a large expenditure of time and patience—yet often success or failure hangs upon the

1. F. A. Davis Co., Philadelphia, 1897. In this work 100 consecutive cases of headache are analysed from the eye-standpoint and the results of eye-treatment are given in full.

2. See preceding table of terms used.

discovery of what a patient cannot voluntarily disclose, and which the oculist has to demonstrate by the judicious use of prismatic glasses. Because one competent observer perchance overlooks or fails to find what another may discover, is no excuse for a wholesale condemnation of a method or a groundless prejudice on the part of a disbeliever.

Each case that presents to the general practitioner obscure nervous symptoms where causation is in doubt, is apt to present also to the oculist as well some "eye problem" that cannot be solved in a minute or possibly in a month. Such patients are often unconsciously and incessantly making the best efforts that they can to overcome the defects that oculists may be looking for in ocular adjustment. These have probably existed from birth, and the patients have been trying all their lives to overcome them. Just as a man unconsciously acquires a gait to make a short leg less apparent when walking, so victims to eye-strain, are often able to simulate a closer approach to "orthophoria" than they really possess. The oculist of the largest experience in the investigation of such cases is the most apt to solve perplexing eye-problems first. I have, for example, worked two months or more over the puzzling eye-tests of some epileptic patient who presented a very complex eye-problem to decipher before I felt sure which muscle of the eye I should first readjust; while in some other cases the line of procedure would become to me equally apparent and perhaps more positive after five minutes of testing the eyes with a plain red glass.

CONCLUSIONS.

As the result of many years of personal investigation respecting the relationship of eye-strain to obscure nervous diseases and phenomena, I would offer the following résumé of my work in connection with headache, neuralgia and nervous prostration:

1. I believe that "eye-strain" is the fundamental and underlying cause of at least 90 per cent. of all headaches and neuralgias; where diabetes, uremia and other forms of toxemia can be eliminated from the list of possible causes.

2. Typical cases of "sick-headaches" in patients under forty years of age are almost invariably due to some type of eye-strain. If developing after the fortieth year, uremia and diabetes frequently are factors in headaches associated with vomiting; although an important eye-factor may co-exist with some form of toxemia (when detected)—chiefly in uncorrected hypermetropia or a loss of the power of accommodation of vision.

3. Headaches are too frequently attributed by medical men to errors of diet, torpidity of the liver, use of tobacco, etc. Cathartics will often abort attacks; and abstinence from tobacco, rich foods, and sweets may for a time decrease the frequency of sick-headaches; but the radical cure of sick-headaches is accomplished in a very large percentage of sufferers from these paroxysms by eye-treatment alone.

Personally, I spent much of my life from the age of four to thirty in bed from uncontrollable paroxysms of headache and vomiting; and when brought at last to a stage of utter physical collapse from unsuspected eye-strain, the correction of my own eye-defects restored me, as if by magic to health and a capacity for labor that I had never before known. My poor abused liver, that had been stirred up by drugs for years to abort the paroxysms of pain, must bless the day that revealed the actual cause; and the use of tobacco, to which many eminent men had attributed my sufferings, is still my solace and delight. At different times in my life my headaches had been diagnosed as due to "nicotine poisoning of the retina," cerebral congestion, fermentation of food, insufficient bile secretion, and many other speculative conditions while the actual cause was discovered by myself almost by accident, and my life's work became an outgrowth of my recovery and restoration to health.

4. The first step that should be taken by all intelligent medical practitioners of the present day in any case of headache, neuralgia or nervous prostration, is unquestionably to have the eyes of the patient examined carefully (by the latest methods) for "latent" or "manifest" errors of refraction and for "latent" anomalies of adjustment of the ocular muscles. Until such investigations are made, time is generally ill-spent in medication, and the sufferings of the patient often needlessly prolonged.

The first and second chapters of my work on Eye-strain, to which I have previously referred, will enable the general practitioner of medicine to make himself sufficiently familiar with eye-examinations either to test his own patients or to confirm the views of those specialists to whom he may refer them for an opinion.

5. No eye examination for refractive errors without the instillation of a mydriatic to dilate the pupils should be considered as final. Valuable as are ophthalmoscopic examinations, the employment of the shadow test, the use of the ophthalmometer of Javal, the reading of Snellen's test-type through trial glasses, etc., no method is absolutely accurate and positive unless the accommodation of the patient is relaxed by some mydriatic.

6. The correction of the refraction of any patient suffering from eye-strain is of vital importance in many instances, before any positive opinions can be formed regarding anomalies of the ocular muscles, it is always important to ascertain if a full refractive correction by glasses, worn constantly for a period, materially modifies or corrects the "manifest" or "latent" heterophoria.

Without a thorough investigation of refractive anomalies in any patient, all muscular tests are peculiarly apt to be open to suspicion and doubt.

7. Oculists do not all think alike. Differences of opinion in diagnosis are usually due, however, to different methods of examination employed; coupled not infrequently, however, with prejudice and undue haste in arriving at conclusions respecting complex eye-problems.

8. I believe that an enormous percentage of cases that are called "*nervous prostration*" by medical men as well as the laity are in reality nervous bankrupts from an incessant leakage of nervous force entailed by some type of eye-strain, that has probably existed from birth and that will continue until death unless scientifically investigated and arrested.

I have seen so many instances of startling recoveries of nervous power almost immediately after a correction of "esophoria" and "hyperphoria[1]," that I cannot too strongly state my convictions here regarding the importance of investigating the eyes of patients who collapse nervously without any apparent organic disease, and who thereafter are kept alive by tonics, travel, massage, electricity, and stimulants because the underlying causes are not properly sought for and rectified.

ERRATA.—The top line of page 655 of the April JOURNAL should have read: "*One of his physicians sent him to the late Dr. Loring, the famous*"

345 MADISON AVENUE, NEW YORK CITY.

